# Rapid and accurate peripheral nerve imaging by multipoint Raman spectroscopy

**DOI:** 10.1038/s41598-017-00995-y

**Published:** 2017-04-12

**Authors:** Yasuaki Kumamoto, Yoshinori Harada, Hideo Tanaka, Tetsuro Takamatsu

**Affiliations:** 1 Department of Pathology and Cell Regulation, Graduate School of Medical Sciences, Kyoto Prefectural University of Medicine, 465 Kajii-cho Kawaramachi-Hirokoji, Kamigyo-ku, Kyoto, 602-8566 Kyoto Japan; 2 Department of Medical Photonics, Graduate School of Medical Sciences, Kyoto Prefectural University of Medicine, 465 Kajii-cho Kawaramachi-Hirokoji, Kamigyo-ku, Kyoto, 602-8566 Kyoto Japan

## Abstract

Raman spectroscopy allows label-free, minimally invasive, and accurate detection of peripheral nerves. However, the conventional Raman imaging technique is time-consuming when measuring a large area of a sample. Establishing a method for rapidly acquiring spatial distribution of a bundle of peripheral nerve fibers is an essential step for Raman spectroscopy towards application in clinical surgery. Here we present a multipoint Raman spectroscopic technique for rapid peripheral nerve imaging. In only 5 seconds, spectra at 32 points situated on *ex vivo* rat peripheral nerve bundles and adjoining connective tissues were acquired. Principal component regression and discriminant analysis of spectra revealed that the sensitivity, specificity and accuracy for nerve detection were 85.8%, 96.0%, and 90.8%, respectively. Of 158 peripheral nerves, 152 (96.2%) showed ratio of the number of nerve-positive prediction points to the total measurement points being 0.4 or larger, whereas 119 (99.2%) connective tissues among 120 showed ratio smaller than 0.4. Based on the ratio and a bright-field image of the sample, accurate visualization of peripheral nerves was implemented. The results indicated that the multipoint Raman spectroscopic technique is capable of rapid and accurate peripheral nerve imaging.

## Introduction

Injury of a bundle of peripheral nerve fibers can cause serious functional deficit of a limb or organ. Nerve-sparing surgery^1–16^ and nerve repair^17–23^ are essential for protecting patients who need radical resection of a tumor from postsurgical functional deficit and recovering patients with traumatic limb injury from functional deficit, respectively. Accurate discrimination between peripheral nerve and nerve-like structures such as connective tissues is the key to the success of these surgical operations. Intraoperative identification of a peripheral nerve has long relied on the surgeon’s eyes and anatomical knowledge. However, surgeons find it hard to identify a thin bundle of peripheral nerve fibers less than 1 mm in thickness. Indeed, a number of patients have experienced postsurgical functional deficits of limbs and organs^5, 7, 8, 10, 12, 15^. A recurrent laryngeal nerve can be identified through electrical stimulation driving vocal cord movement in neck and thyroid surgeries^24–26^, but this technique can only detect a bundle containing motor nerve fibers and not one mostly composed of autonomic and/or sensory nerve fibers. Furthermore, it carries a risk of nerve injury due to excess current^25^.

In the most recent decade, several techniques have been applied for visualizing peripheral nerves, for example, by using fluorescent peptides specifically labeling peripheral nerves^27, 28^, spontaneous Raman scattering^29, 30^, coherent anti-Stokes Raman scattering^31^, and combination of autofluorescence and second harmonic generation^32–34^. Among these, the spontaneous Raman scattering technique has several advantages for clinical use; it does not require tissue pretreatment such as staining, allowing use the in human body. Moreover, the tissue-type discrimination of the Raman technique is reliable since Raman scattering is an intrinsic fingerprint of a tissue and reflects differences in molecular composition among different tissues^29, 30^. Indeed, our group has previously shown that the Raman technique can discriminate peripheral nerves from their adjacent tissues including blood vessels, adipose tissue, skeletal muscle, and fibrous connective tissues^29^. For *ex vivo* detection of rat peripheral nerve, the sensitivity, specificity, and accuracy of the Raman technique were 94.2%, 92.0%, and 92.9%, respectively^30^. In addition, the spontaneous Raman technique allows relatively safe nerve detection, since excitation of spontaneous Raman scattering does not require high intensity laser as the coherent Raman scattering technique does.

The essential step of the spontaneous Raman technique towards clinical use is to establish a technique for rapidly measuring the spatial distribution of thin bundles of peripheral nerve fibers. Combination with a laser-scanning technique can measure the spatial distribution of tissues, but it will require a long acquisition time^35, 36^ since the efficiency of Raman scattering is tremendously low. The slit-scan Raman imaging technique using line illumination allows short-time acquisition of a tissue Raman image by reducing the number of scans^37–39^, but this technique still requires several minutes or more, which is too long a waiting time for a surgical procedure, for two-dimensional imaging.

Here we suggest multipoint Raman spectroscopy^40–42^ for rapid imaging of a bundle of peripheral nerve fibers. The multipoint Raman spectroscopic technique enables rapid acquisition of a number of spectra over a large area of a sample. Together with the multipoint Raman technique, we also suggest use of a bright-field image, where tissue structures of candidates for nerve bundles can be quickly revealed based on morphological information. For identifying and visualizing nerve bundles, concurrent analysis of multipoint Raman spectra with a corresponding bright-field image is executed. The usefulness of the presented technique is evaluated by discriminating peripheral nerves and adjacent connective tissues, which visually resemble each other.

## Results

### Multipoint spontaneous Raman spectral mapping

**Figure 1:**
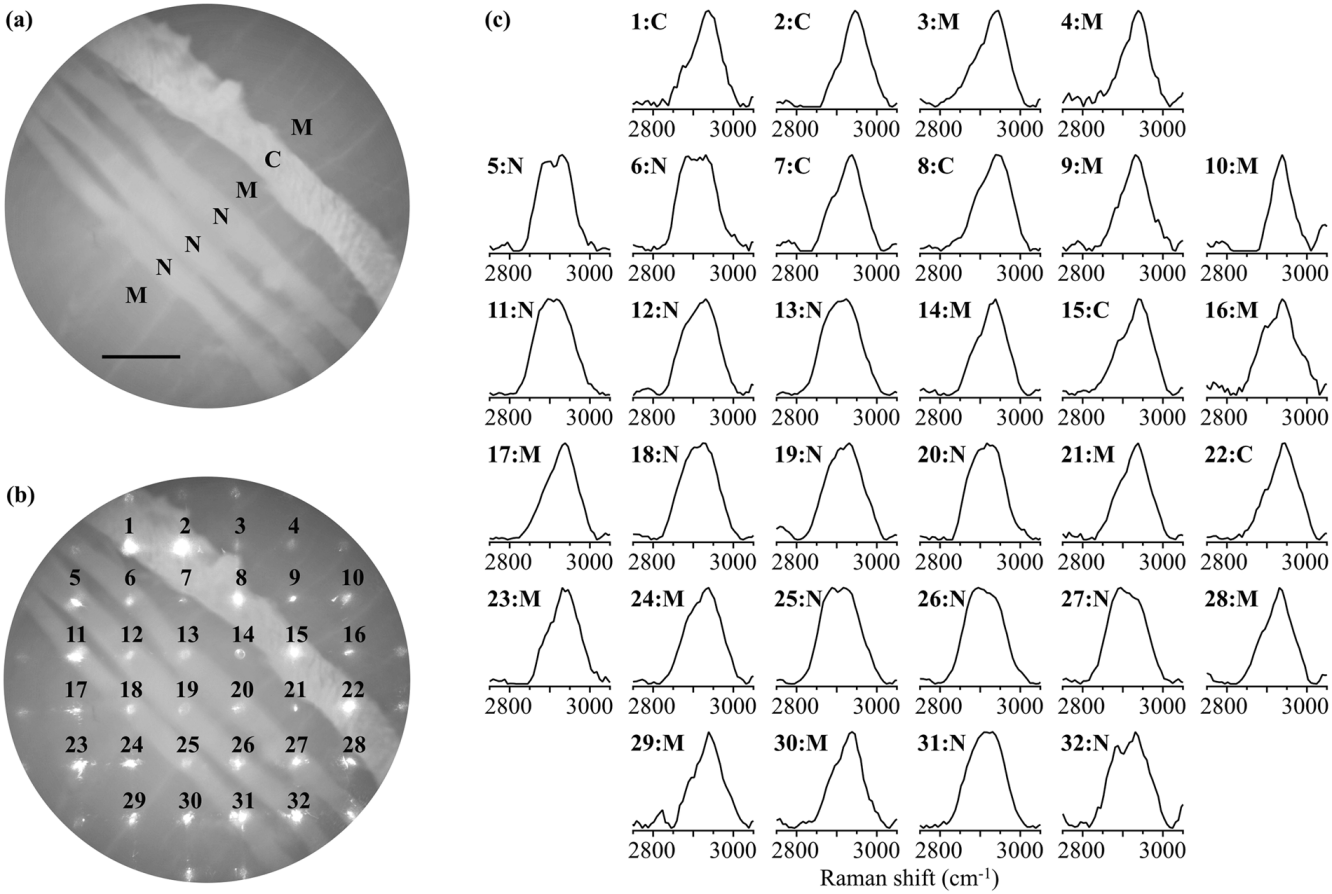
(**a**) Bright-field image of a test sample containing bundles of peripheral nerve fibers (N), connective tissue (C), and a skeletal muscle tissue (M). The scale bar is 1 mm. (**b**) Bright-field image of the sample together with multiple excitation laser irradiation spots. (**c**) Raman spectra obtained at the laser spots seen below numbers in (**b**). The signal accumulation time was 5 seconds. N, C, and M indicate the tissue type at each spot position. The arrowheads indicate two characteristic bands at 2895 and 2931 cm^−1^. The spectra were preprocessed.

We measured a test sample to evaluate if the multipoint Raman spectral mapping technique (see Methods or for details) is useful for rapidly discriminating a bundle of peripheral nerve fibers and another type of tissue resembling the nerve bundle. As a test sample, we used a thin nerve bundle and connective tissue on a skeletal muscle tissue, which mimics a tissue visible to the eyes in surgery. Figure 1 presents a dataset acquired by multipoint Raman spectral mapping and bright-field imaging of a test sample. In the bright-field image, four nerve-like structures are seen. Three relatively thin structures located in parallel at the middle are nerve bundles with the thickness of ~0.4 mm, and the thicker structure at the upper right is connective tissue with the thickness of ~0.6 mm. Identification of nerve bundles by eyes is difficult. Shown in Fig. 1(b) is the bright-field image of the sample irradiated with multiple laser spots for Raman excitation. The circular spots below numbers (1, 2, 3, …, 32) correspond to Raman measurement points. Figure 1(c) shows 32-point individual Raman spectra obtained for 5-second signal accumulation time. The spectra show two characteristic bands at 2895 and 2931 cm^−1^, as indicated by arrowheads. The 2895 cm^−1^ band is remarkable for almost all the spectra obtained at the nerve (points 5, 6, 11–13, 18–20, 25–27, 31, 32). This band can be assigned to CH_2_ asymmetric stretching mode, which is known to be particularly intense for a bundle massively containing myelinated nerve fibers^29, 30^. On the other hand, the intense 2931 cm^−1^ band is seen in all the spectra. This band can be assigned to CH_3_ symmetric stretching mode and is known to be intense for nerve, connective tissue, and skeletal muscle tissue^29, 30^. For clarity of spectral difference among these three types of tissues, overlay of average spectra of the peripheral nerves, connective tissue, and skeletal muscle is shown in Fig. S2. Although the 2855 cm^−1^ band in previous studies^29, 30^ is not seen here, it could be merged into the 2895 cm^−1^ band due to low spectral resolution of the instrument.

### Tissue-type discrimination at individual Raman measurement points

**Figure 2:**
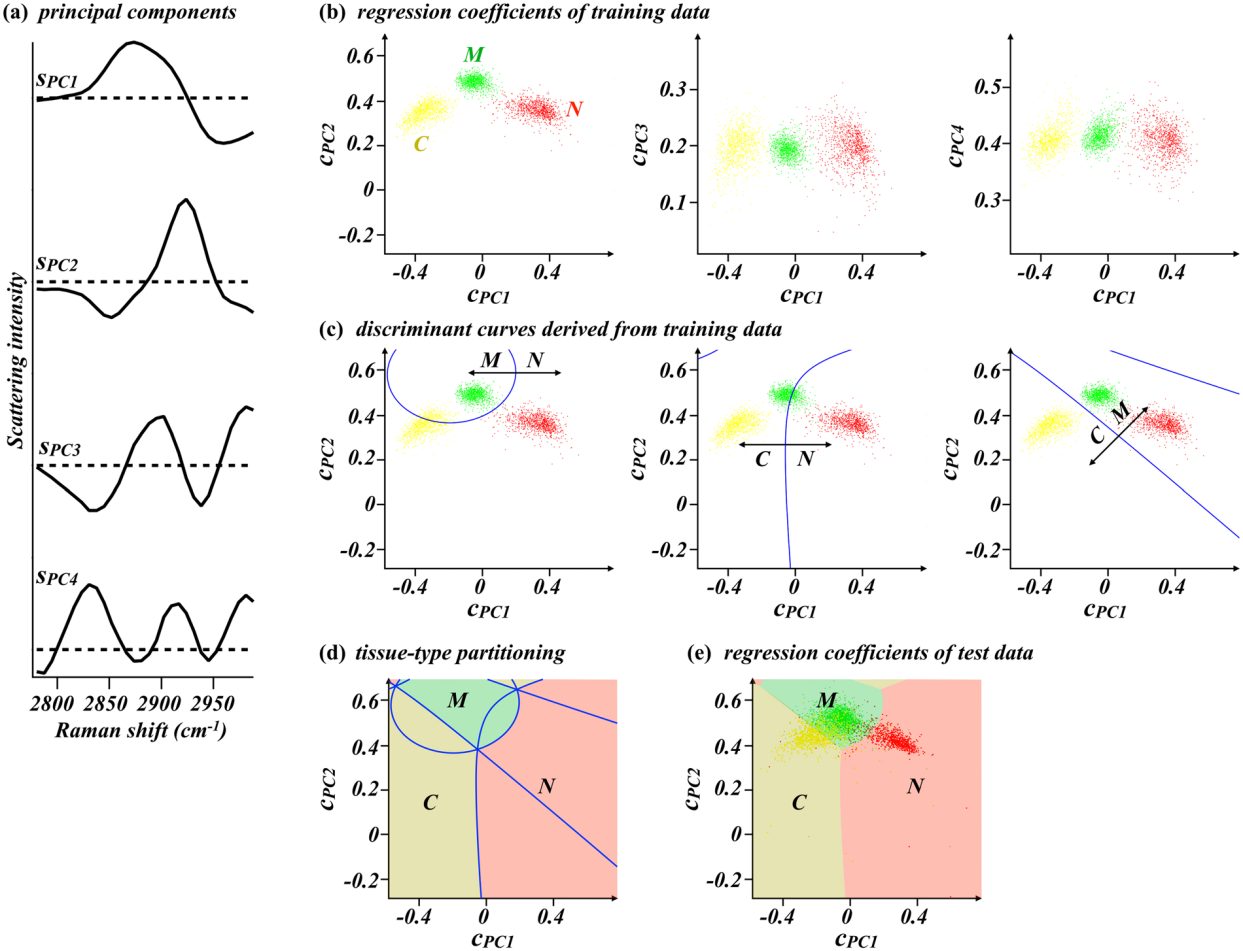
(**a**) The first to fourth principal components of training dataset composed of 1000 peripheral nerve spectra, 1000 connective tissue spectra, and 1000 skeletal muscle tissue spectra (*s*_*PC1*_ to *s*_*PC4*_). Horizontal dashed lines indicate zero intensity for individual principal components. (**b**) Principal component regression coefficient plots of the training spectra. *c*_*PC1*_, *c*_*PC2*_, *c*_*PC3*_, and *c*_*PC4*_ are regression coefficients to the four principal components shown in (**a**) (*s*_*PC1*_, *s*_*PC2*_, *s*_*PC3*_, and *s*_*PC4*_, respectively). (**c**) Discriminant curves discriminating the Raman spectra groups of peripheral nerve from skeletal muscle tissues (left), peripheral nerve from connective tissues (middle), and connective tissues from skeletal muscle tissues (right) in the space of *c*_*PC1*_ to *c*_*PC2*_ plot. (**d**) A graph overlaying all the discriminant curves in (**c**) and three distinct areas assigned to nerve-positive prediction region (light red), connective-tissue-positive prediction region (dark yellow) and muscle-positive prediction region (light green). Individual areas were derived from the M/N and C/N discrimination curves, the C/N and C/M discrimination curves, and the M/N and C/M discrimination curves in (**c**), respectively. (**e**) Results of principal component regression of the 3840 test spectra with the four principal components shown in (**a**). The tissue-type partitions are overlaid. N: peripheral nerve bundle. C: connective tissue. M: skeletal muscle tissue.

To quantitatively discriminate tissue type at individual Raman measurement points, we employed principal component regression and discriminant analysis, an analytical method powerful for discriminating data groups with multicollinearity^30, 43^. The first to fourth principal components (*S*_*PC1*_, *S*_*PC2*_, *S*_*PC3*_, *S*_*PC4*_) of training data composed of nerve, connective, and muscle tissue spectra (n = 1000 for each) are shown in Fig. 2(a). Shown in Fig. 2(b) are results of regression of the training data with the 4 principal components, being two-dimensional plots of regression coefficients (*c*_*PC1*_ vs *c*_*PCm*_: m = 2,3,4). Clearly, individual Raman spectra represented by individual dots for nerve, connective, and muscle tissues are well discriminated along the *c*_*PC1*_ axis. In addition, along the *c*_*PC2*_ axis, spectra of peripheral nerve bundles and connective tissues can also be discriminated from those of skeletal muscle tissues. On the other hand, the three groups cannot be discriminated along the *c*_*PC3*_ and *c*_*PC4*_ axis. Thus, we found that the two-dimensional plot of *c*_*PC1*_ to *c*_*PC2*_ was useful for tissue type discrimination. To quantitatively discriminate individual Raman spectra into corresponding tissue types, discriminant analysis was performed. The resultant curves discriminating each two spectra groups (i.e., N from M, N from C, and C from M) are individually presented in Fig. 2(c). The curves overlaid on the graph have enabled us to assign the space into nerve-positive, connective-tissue-positive, and muscle-positive prediction regions, as shown in Fig. 2(d).

In total, 3840 spectra (N: 851, C: 821, M: 2168) were measured from 120 test samples. The overlay of principal component regression coefficients of the test spectra and the tissue discrimination regions are shown in Fig. 2(e). Among the 3840 test spectra, 814, 699, and 2327 spectra were predicted as nerve, connective tissue, and muscle, respectively. Table 1 summarizes the results of tissue-type discrimination. Of 851 spectra obtained at the nerve bundles, 730 were truly predicted as nerve, deriving the nerve detection sensitivity of 85.8%, and 788 out of 821 spectra at connective tissues were predicted as connective tissue or muscle with the nerve detection specificity of 96.0% on connective tissues. The nerve detection accuracy is thereby calculated to be 90.8%.

**Table 1:** Results of tissue-type discrimination of the 3840 test spectra.

	N (n = 851)	measured tissue		
		C (n = 821)	M (n = 2168)	
discrimination result	N (n = 814)	730	33	51
	C (n = 699)	18	578	103
	M (n = 2327)	103	210	2014

N: peripheral nerve tissue. C: connective tissue. M: skeletal muscle tissue.

### Tissue-type mapping and its overlay with a bright-field image

**Figure 3:**
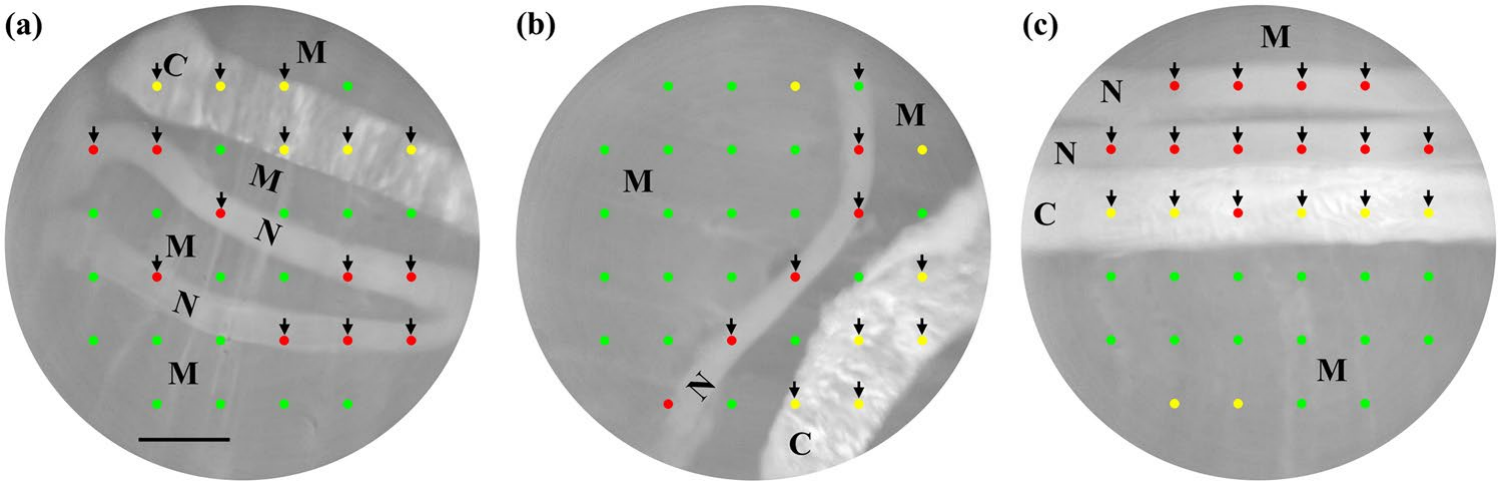
Representative results of tissue-type mapping overlaid with bright-field images. Colored circular spots exhibit Raman measurement points, that is, tissue-type discrimination points. At red, yellow, and green points, tissues are discriminated as peripheral nerve bundle, connective tissue, and skeletal muscle tissue, respectively. The scale bar is 1 mm. N, C and M denote myelinated nerve bundle, connective tissue and skeletal muscle tissue, respectively.

Representative results of tissue-type mapping, which was implemented by overlaying tissue-type discrimination results at individual Raman measurement points and a corresponding bright-field image, are shown in Fig. 3. In Fig. 3(a), two relatively thin structures located at the middle are nerve bundles, while the relatively thick structure at the top is connective tissue. These tissues were measured at 15 points indicated by arrowheads. Nerve-positive prediction points and connective-tissue-positive prediction points only appear at peripheral nerve and connective tissue, respectively, meaning that tissue type discrimination of this sample is completely accurate. However, this is not the typical case, but false positive and/or negative predictions often arise in many cases. In Fig. 3(b), a relatively thin structure located at the middle is a peripheral nerve tissue, while the relatively thick structure at the bottom right is connective tissue. The nerve bundle and connective tissue were measured at 10 points indicated by arrowheads. The point indicated by the arrowhead at the top is falsely discriminated as skeletal muscle tissue. In Fig. 3(c), the upper two relatively thin structures are peripheral nerves bundles, while the relatively thick structure at the middle is connective tissue. The peripheral nerve bundles and connective tissue were measured at 16 points indicated by arrowheads. The center-left point on the connective tissue is falsely discriminated as peripheral nerve bundle.

### Peripheral nerve detection using the ratio of the number of nerve-positive prediction points to the total measurement points

**Figure 4:**
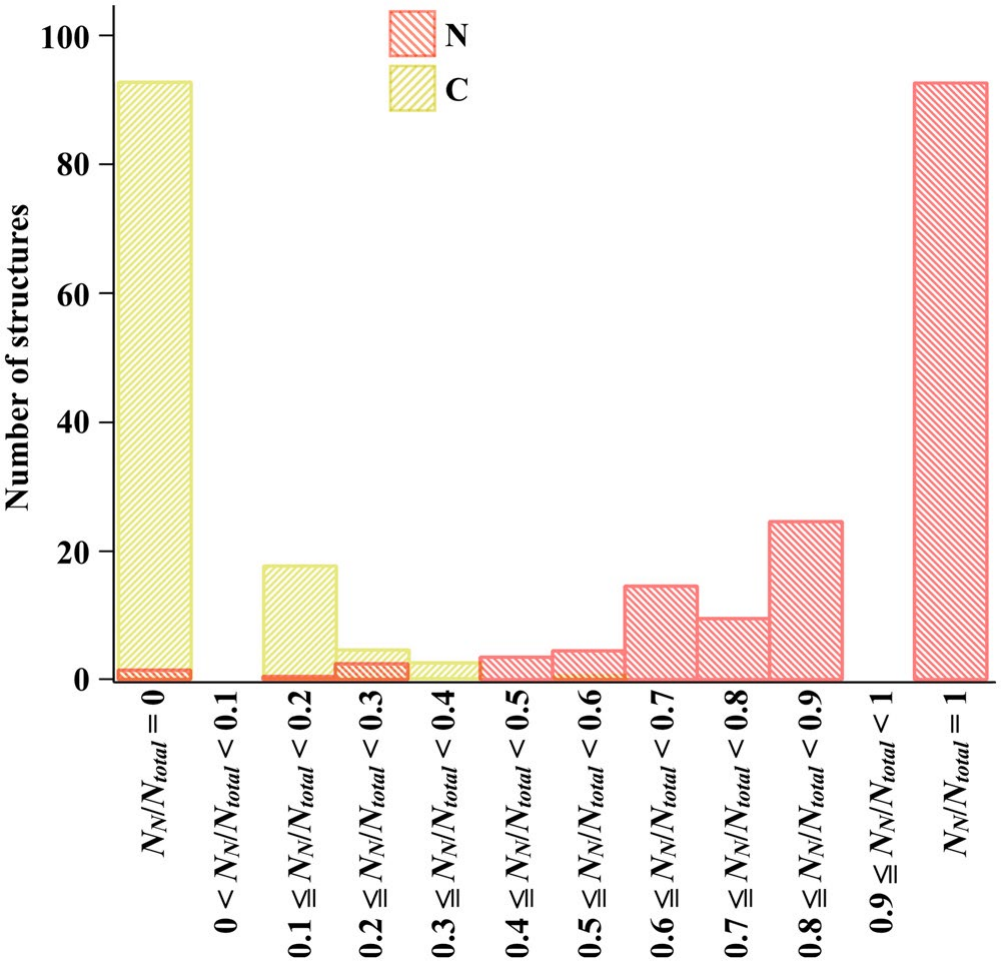
Histogram summarizing the ratio of the number of nerve-positive prediction points (*N*_*N*_) to that of total Raman measurement points (*N*_*total*_) at individual nerve bundles (N: red) and connective tissues (C: yellow). The total number of analyzed peripheral nerve and connective tissues is 158 and 120, respectively.

We counted the number of nerve-positive prediction points (*N*_*N*_) on individual peripheral nerves and connective tissues (Fig. S3). Of 160 measured nerve bundles, 144 contained three or more nerve-positive prediction points, whereas only 1 of the measured connective tissues contained three or more nerve-positive prediction points. Peripheral nerve and connective tissues can be discriminated at the threshold of *N*_*N*_ = 2 with high sensitivity, selectivity, and accuracy. However, *N*_*N*_ is not necessarily a reliable index for predicting the type of a given tissue since the total number of Raman measurement points on individual peripheral nerve and connective tissues are not always constant. The ratio *N*_*N*_/*N*_*total*_ seems to be a better index. *N*_*N*_/*N*_*total*_ of all the measured peripheral nerve and connective tissues are shown in Fig. 4. Among 160 nerve bundles, 2 were removed from analysis since they each had a single Raman measurement point and were no longer included in the results of multipoint Raman spectroscopy. Table 2 summarizes the number of true positive, false positive, true negative, and false negative nerve predictions of individual tissues when various thresholds of *N*_*N*_/*N*_*total*_ are set to discriminate peripheral nerve from connective tissues. When tissues with *N*_*N*_/*N*_*total*_ = 0.4 or larger are determined as peripheral nerve, 96.2% of analyzed nerve tissues and 99.2% of analyzed connective tissues are successfully discriminated, deriving the best tissue discrimination accuracy, 97.5%.

**Table 2:** Results of peripheral nerve detection using *N*_*N*_/*N*_*total*_.

lowest *N*_*N*_/*N*_*total*_ for nerve detection	0	0.1	0.2	0.3	0.4	0.5	0.6	0.7	0.8	0.9	1
TP	158	156	155	152	152	148	143	128	118	93	93
FN	0	2	3	6	6	10	15	30	40	65	65
TN	0	93	111	116	119	119	120	120	120	120	120
FP	120	27	9	4	1	1	0	0	0	0	0
Sensitivity (%)	100	98.7	98.1	96.2	96.2	93.7	90.5	81.0	74.7	58.9	58.9
Specificity (%)	0	77.5	92.5	96.7	99.2	99.2	100	100	100	100	100
Accuracy (%)	56.8	89.6	95.7	96.4	97.5	96.0	94.6	89.2	85.6	76.6	76.6

TP: true positive. FN: false negative. TN: true negative. FP: false positive.

Note: The average and the standard deviation of *N*_*total*_ for the total 278 tissues were 6.0 and 1.9, respectively.

**Figure 5:**
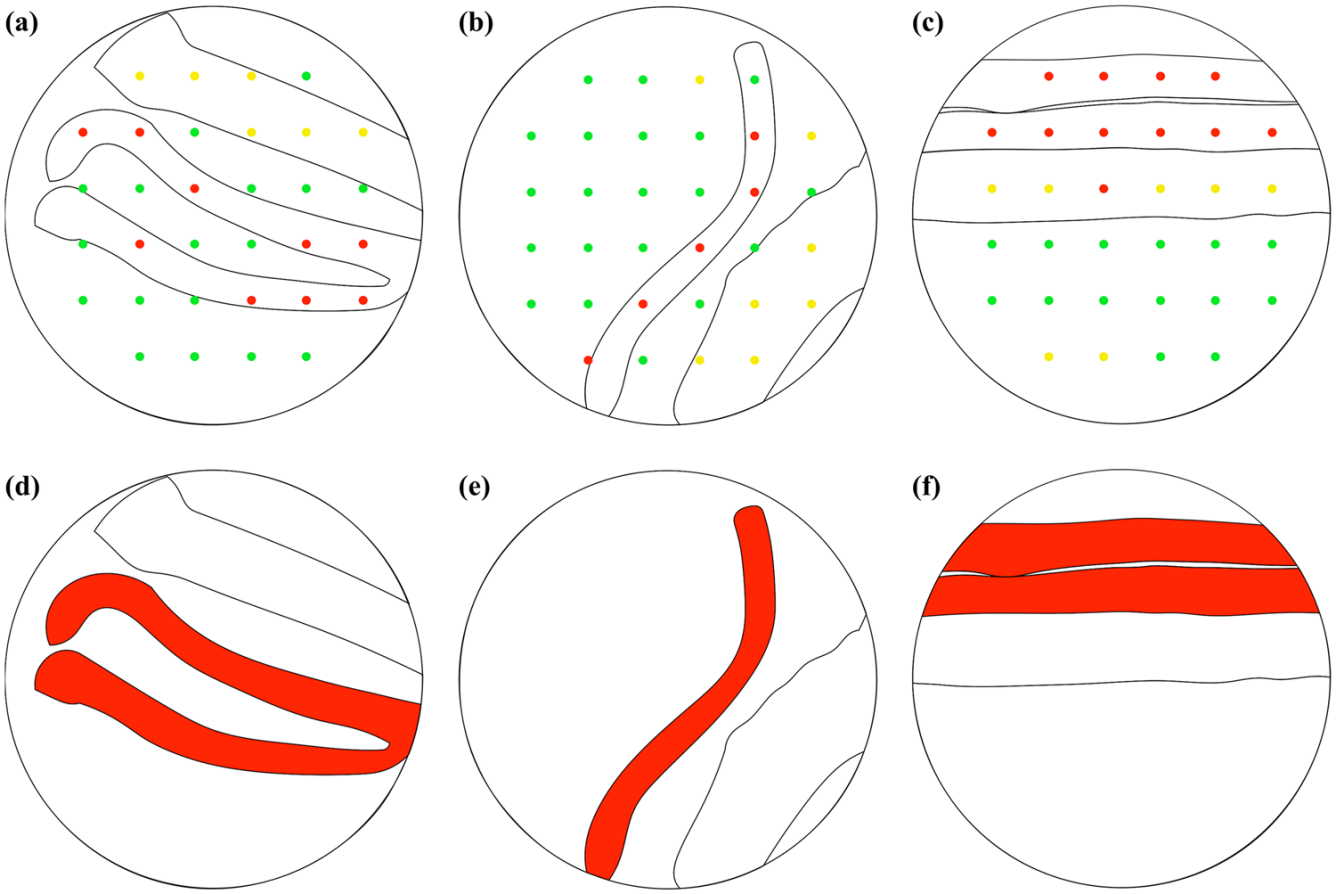
Visualizing the nerve bundles by morphology-highlighted images, the tissue type maps, and the nerve detection criterion on the *N*_*N*_/*N*_*total*_. (**a**–**c**) The overlay of morphology-highlighted images, and tissue type maps of samples shown in Fig. 3. At red, yellow, and green points, tissues are discriminated as peripheral nerve, connective, and skeletal muscle tissues, respectively. (**d**–**f**) The nerve bundles highlighted in red according to the nerve detection criterion of *N*_*N*_ */N*_*total*_ = or >0.4.

Then we combined the tissue type discrimination results based on the ratio with morphological information obtained from bright-field images so that bundles of peripheral nerve fibers in samples are visualized. The overlay of morphology-highlighted images and the tissue type maps for samples shown in Fig. 3 are presented in Fig. 5(a–c). Tissue-type discrimination based on the criterion of *N*_*N*_/*N*_*total*_= or >0.4 successfully visualized the nerve bundles in samples, as shown in Fig. 5(d–f).

## Discussion

The multipoint Raman spectroscopic technique has enabled rapid acquisition of a number of Raman spectra over a sample containing peripheral nerve bundle and connective tissue. Tissue-type mapping through discriminant analysis of Raman spectra, followed by its overlay with a bright-field image, and tissue discrimination using *N*_*N*_/*N*_*total*_ allowed accurate imaging of peripheral nerve bundles. We found that peripheral nerve discrimination from connective tissue by using the presented technique is better than that at individual Raman measurement points, i.e., sensitivity: 96.2%; specificity: 99.2%; accuracy: 97.5% for the presented technique vs sensitivity: 85.8%; specificity: 96.0%; accuracy: 90.8% for the single-point measurement. The tissue discrimination results at individual points are identical to those obtained from single-point Raman measurement of a tissue.

The threshold value for the most accurate peripheral nerve detection in this study is *N*_*N*_/*N*_*total*_ = 0.4. However, we should note that threshold value depends on the sample and/or measurement conditions change. How to determine the threshold value of *N*_*N*_/*N*_*total*_ is the key of the presented technique. The quasi-optimal value can be estimated from the sensitivity and specificity of peripheral nerve detection at individual Raman measurement points. Of the total Raman measurement points at connective tissues 4.0% were nerve-positive prediction points since the nerve detection specificity of the overall individual points was 96.0%, whereas 85.8% (=the nerve detection sensitivity) of the total Raman measurement points at peripheral nerve tissues were nerve-positive prediction points. It is expected that peripheral nerve and connective tissues can be well discriminated when the threshold value of *N*_*N*_/*N*_*total*_ is set at the average of these two values (i.e. (0.04 + 0.858)/2 = 0.449). Setting *N*_*N*_/*N*_*total*_ = 0.449 as the threshold value for detecting peripheral nerves derived the nerve detection sensitivity, specificity, and accuracy of 93.7%, 99.2%, and 96.0%, respectively. This result is close to the result obtained with the optimal threshold value of *N*_*N*_/*N*_*total*_ = 0.4 (i.e. sensitivity: 96.2%; specificity: 99.2%; accuracy: 97.5%).

Raman spectroscopic detection of peripheral nerve tissues may also be conducted by means other than *N*_*N*_/*N*_*total*_. For example, cancer was reportedly discriminated by a mean spectrum over the tissue^42, 44–46^. As shown in Table S1 for mean spectra of individual tissues, the sensitivity, specificity, and accuracy for nerve detection were 93.7%, 99.2%, and 96.0%, respectively, values superior to those of the single-point measurement. This is because averaging derives a spectrum with higher signal-to-noise ratio than the original individual spectra. The sensitivity for nerve detection using a mean spectrum, on the other hand, is inferior to that for the present method using *N*_*N*_/*N*_*total*_, indicating that a surgeon may cut a peripheral nerve by misidentification with the mean modality more frequently than the ratio one during surgery. Additionally, a mean spectrum tends to be affected by a measurement point giving an intense signal, and therefore, tissue discrimination using the mean spectrum can be ruined by a single measurement point emitting an intense false signal (i.e. adipose-tissue-like Raman spectrum at a peripheral nerve) and/or intense autofluorescence. Contrarily, the advantage of this method is that there is no need for optimizing the threshold value, that is, simplicity. In surgery, combination of these two analytical methods, which can compensate for the drawbacks of each other, may assist in reliable, accurate detection of peripheral nerves.

This study used Raman spectra accumulated as quickly as in 5 seconds. The readout time of the detector, the calculation time of data preprocessing, principal component regression, discriminant analysis, tissue-type mapping, and bright-field imaging in total took about three seconds. For switching Raman mapping and bright-field imaging, highlighting morphological information from a bright-field image, counting *N*_*N*_ and *N*_*total*_ on individual tissues, and tissue-type discrimination of individual peripheral nerve and connective tissues, our manual operations were time-consuming. These operations, when automated, will take a few seconds. Overall, peripheral nerve detection using the present technique will be complete in around 10 seconds.

The presented multipoint Raman spectral mapping and following analyses have some other advantages over the single-point Raman measurement of a tissue. Illumination at the boundary of structures may generate a mixture spectrum of two different tissues, leading to inconstant and inaccurate tissue prediction. Peripheral nerve detection using the multipoint Raman technique and the following analysis using *N*_*N*_/*N*_*total*_ is not necessarily affected by a spectrum at the boundary, because it can eliminate the influence of the ambiguous spectrum by thresholding.

Finally, we describe the limitations of the present technique. First, thin peripheral nerve bundles and nerve-bundle-like structures often can be out of illumination. Indeed, 4 peripheral nerve bundles out of 160 and 1 connective tissue out of 120 had only 1 or 2 Raman measurement points in this study. Thinner structures (i.e. 0.1–0.2 mm in thickness) will be frequently out of illumination with the instrument used. For detection of such a thin nerve bundle, narrow spacing between illumination spots is preferred, but just narrowing spaces may decrease the field of view of measurement unless the number of illumination spots is increased. However, the maximum number of illumination spots allowable is limited to the pixel number of the detector array of a camera installed in the spectrograph along the long axis of the entrance slit of the spectrograph (*N*_*pixel*_), i.e., 400 for a 1340 × 400 pixels CCD camera). When the diameter of detection fibers (*D*_*fiber*_) is larger than the pixel length (*L*_*pixel*_), this could be *N*_*pixel*_ × *L*_*pixel*_ */(D*_*fiber*_ + *S*_*fiber*_ *)*, where *S*_*fiber*_ is the separation between fibers arranged along the slit. At present, the maximum number of illumination spots allowable is 60, and the illumination pattern of an 8 × 8 square lattice with 60 illumination spots (excluding corners) and 0.4 mm separation between neighboring spots, covering the field of view of 3.4 mm, is implementable. This illumination pattern, which can be good enough for detecting thin nerve bundles, will resolve the issue. Second, accuracy of tissue prediction at individual Raman measurement points can depend on measurement objects and conditions. For instance, *in situ* measurement will have more false positive or negative prediction due to other substances (e.g. blood, body fluid) than *ex vivo* measurement. The tissue prediction accuracy also depends on the amount of myelin in a bundle^29, 30^. Thickness of a nerve bundle may also affect the prediction accuracy. These factors can be eliminated by optimizing the threshold value of *N*_*N*_/*N*_*total*_ for individual measurement objects and conditions. Finally, some multiple illumination spots cannot properly be focused at target tissue when the surface of the tissue is not orthogonal to the optical path axis or the surface has a degree of roughness. Gently pushing the surface with a flat coverslip with liquid immersion made the sample surface relatively flat and orthogonal to the optical path axis adequately enough for proper focusing of multiple illumination spots. We have developed a multipoint Raman measurement probe with the head covered by a flat glass plate (patent pending). Despite these limitations, the present multipoint Raman spectral mapping and following analyses will be useful for accurate and rapid detection of thin bundles of peripheral nerve fibers during surgery.

## Methods

### Animals

All animal experiments were conducted with the approval of and in accordance with guidelines from the Committee for Animal Research, Kyoto Prefectural University of Medicine. Adult male Wistar rats (11–17 weeks, n = 4) with a weight of 310–480 g were analyzed. Animals were purchased from Shimizu Laboratory Supplies Co. Ltd. (Kyoto, Japan). After euthanasia of a rat, surgical operation was started; 0.5 ml heparin sodium solution (Ajinomoto, Co. Inc.) was injected into the inferior vena cava. Bundles of sciatic nerve, facial nerve, saphenous nerve, intercostal nerves, and celiac nerves, leg tendons (i.e. Achilles tendon), and femoral skeletal muscle tissues were sampled. The excised tissues were rinsed with physiological saline (0.9% NaCl aqueous solution), and were kept in physiological saline on ice until measurement.

### Test sample

As a test sample, peripheral nerve and connective tissues located on skeletal muscle tissues were used. Nerve tissues were fresh excised intercostal nerve, saphenous nerve, or femoral nerve, which are mostly composed of myelinated nerve fibers. The average diameter is 0.5 mm with the standard deviation of 0.18. Connective tissues were fresh excised tendons cut into thin structures mimicking thin connective tissues, which are hard to discriminate from peripheral nerve during surgery. The average diameter of the slimmed tendons was 0.98 mm with the standard deviation of 0.24. Skeletal muscle tissues were fresh excised femoral muscle blocks cut to around 2 cm × 2 cm. A whole sample was placed on a microscope slide. To avoid drying, tissues were immersed in physiological saline and covered with a coverslip (No.1; Matsunami Glass, Inc.). The total number of analyzed nerve bundles, thin connective tissues, and skeletal muscle tissue blocks was 160, 120, and 4, respectively.

The type of a given tissue in a test sample where we located known nerve and connective tissue on a skeletal muscle tissue was judged by the position and morphology.

### Training sample

Fresh excised bundles of sciatic nerve, saphenous nerve, and facial nerve, which are mostly composed of myelinated nerve fibers, leg tendons, and femoral skeletal muscle tissue blocks were placed on a microscope slide as a training sample of peripheral nerve, connective, and skeletal muscle tissues, respectively. To avoid drying, tissues were immersed in physiological saline and covered with a coverslip (No.1; Matsunami Glass, Inc.).

### Multipoint Raman spectral measurement

A single-frequency diode-pumped solid state laser with the wavelength of 532 nm and the output of 300 mW (DPSS laser series, LASOS Lasertechnik) was used as Raman excitation light source. A multimode optical fiber (Thorlabs) transferred the laser beam to the apparatus body. A microlens array (Edmund Optics) generated multiple excitation laser spots at a conjugate plane of the sample plane. The multiple laser spots were projected to the tissue surface by a couple of lenses including an achromatic doublet lens with NA = 0.34 as the objective lens. The sample irradiation pattern was a 6 × 6 square lattice with 32 irradiation spots (excluding corners) and the spot separation of 0.7 mm, covering the field of view of 4.1 mm. The total laser power of 32 irradiation spots was 27–28 mW as measured with Si photodiode (Thorlabs). Each irradiation spot had the diameter of 50 µm. Backward Stokes Raman scattering from a sample was separated from the excitation beam by a shortwave pass dichroic mirror (Edmund Optics). The remaining Rayleigh scattering component was removed by a longwave pass edge filter (Edmund Optics). Raman scattering was focused by an f = 20 mm imaging lens into 32 fibers with the diameter of 100 µm. One fiber collected Raman scattering generated at one sample irradiation spot. The fibers at the other side were rearranged with a separation of 10 µm in a line parallel to the longer axis of the entrance slit of a spectrograph (Holospec f/1.8, Andor Technology), so that the Raman scattering from different fibers was independently imaged on a CCD camera (Newton 920, Andor Technology) mounted to the spectrograph. The pixel size of the detector (26 µm x 26 µm) is much smaller than the fiber diameter so that Raman scattering from individual spots was not significantly mixed at a pixel of the detector. The spectrograph was equipped with a transmission grating (HS-HFG-650, Andor Technology) providing the spectral resolution of 7 cm^−1^. Raman spectra of individual Raman measurement points were results of binning 3 or 4 pixels at the detector in the direction of the longer axis of the slit.

See Fig. S1 for the schematic of the multipoint Raman setup.

### Bright-field imaging

For bright-field imaging, a sample was illuminated from every direction with four LEDs surrounding the sample. The scattering light was collected with the same objective lens as used for Raman scattering excitation and collection, and guided to a CMOS camera (Thorlabs) installed in the Raman measurement system. The field-of-view of the bright-field image is 5.2 mm.

### Spectral preprocessing

Data preprocessing was performed on MATLAB (Mathworks). The high wavenumber region containing high signal-to-noise ratio Raman signals (2563–3204 cm^−1^) was used. All the measured Raman spectra were smoothed by taking moving average of 5 pixels. Baseline in each spectrum was corrected with subtraction of fluorescent background, which was numerically derived by 100-times iterative, 8th-order alternative weighted polynomial fitting with the fitting weight of 0 for 2816–2988 cm^−1^ and 1 for 2563–2816 and 2988–3204 cm^−1^ to the high wavenumber region of each spectrum. The 2780–2988 cm^−1^ region was used for discriminant analysis. Before discriminant analysis, each spectrum was normalized by its euclid norm.

### Principal component regression and discriminant analysis

For discriminating tissue type at each Raman measurement point, principal component regression and discriminant analysis^30, 43^ was utilized. A discrimination model was constructed from 3000 training spectra sets, including 1000 spectra acquired from excised sciatic, facial, and saphenous nerves, 1000 spectra acquired from excised femoral muscle tissue, and 1000 spectra acquired from excised leg tendons. The model can discriminate a tissue based on regression coefficient *C*_*PC*_ = (*c*_*PC1*_, *c*_*PC2*_, …, *c*_*PCn*_), which is calculated from the following equation:

1 $${C}_{PC}={S}_{sample}\cdot {{S}_{PC}}^{T}\cdot {({S}_{PC}\cdot {{S}_{PC}}^{T})}^{-1}$$

where *S*_*sample*_ is a Raman spectrum measured from a sample, *S*_*PC*_ is a spectra set composed of arbitrary Eigen vectors which are obtained from principal component analysis of the 3000 training spectra set. For the 3000 training spectra, *S*_*PC*_ = (*s*_*PC1*_, *s*_*PC2*_), where *s*_*PCi*_ is the *i*th Eigen vector, gave the tissue prediction results with the 99.6% accuracy through the leave-one-out-cross-validation method and quadratic discrimination algorithm.

Principal component analysis and discriminant analysis were performed on MATLAB. Principal component analysis used “pca” function. The discriminant analysis used “fitcdiscr” function for making the discrimination model out of the training spectra set and “classify” function for discriminating sample spectra set. Drawing discrimination curves used “fitcdiscr” function.

### Tissue-type mapping

For tissue-type mapping based on measured Raman spectra and following principal component regression and discriminant analysis, the morphology of tissues on sample is first highlighted. One of the authors (Y. K.) carefully traced the boundary of tissue structures by using computer software. Then, the spatial coordinates of the center of individual laser spots were determined from a bright-field image of a sample irradiated with multiple excitation laser spots.

After the tissue morphology was highlighted, the tissue discrimination results were overlaid with the bright-field and morphology-highlighted images.

Finally, Y. K. carefully counted the numbers of Raman measurement points and nerve-positive prediction points on individual tissues. The total number of Raman measurement points on a given peripheral nerve bundle varies between 1 and 13, with the average of 5.4 and the standard deviation of 1.7. The total number of Raman measurement points on a given connective tissue varies between 3 and 15, with the average of 6.8 and the standard deviation of 1.9.

## Electronic supplementary material

Supplementary Figures and Table
