# Supplementary material for: Rapid and accurate peripheral nerve imaging by multipoint Raman spectroscopy
Source: Sci Rep. 2017 Apr 12;7:845. doi: 10.1038/s41598-017-00995-y (PMC5429797; doi:10.1038/s41598-017-00995-y)
Supplement: Supplementary file 1 — Supplementary Figures and Table [file 41598_2017_995_MOESM1_ESM.pdf]

Rapid and accurate peripheral nerve imaging by multipoint Raman spectroscopy

Yasuaki Kumamoto, Yoshinori Harada, Hideo Tanaka, Tetsuro Takamatsu

Supplementary materials

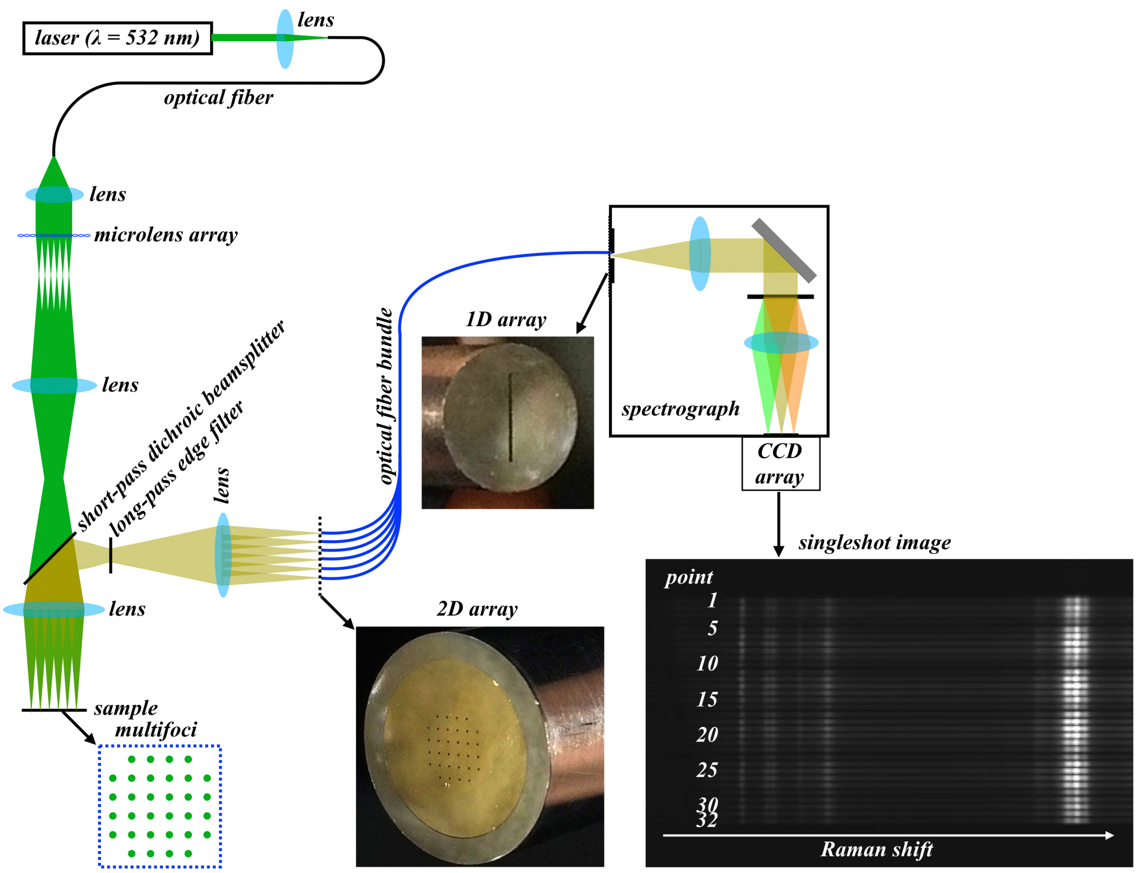

Fig. S1. Schematic of the multipoint Raman spectral mapping setup. Detailed explanation is presented in the Methods section.

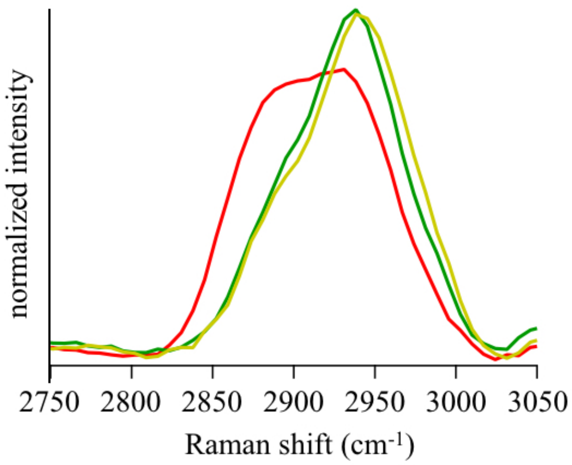

Fig. S2. Overlay of Raman spectra obtained from peripheral nerve bundles (red), connective tissues (yellow), and skeletal muscle tissues (green). The spectrum of each tissue type is an average of spectra measured at each tissue shown in Fig. 1(c).

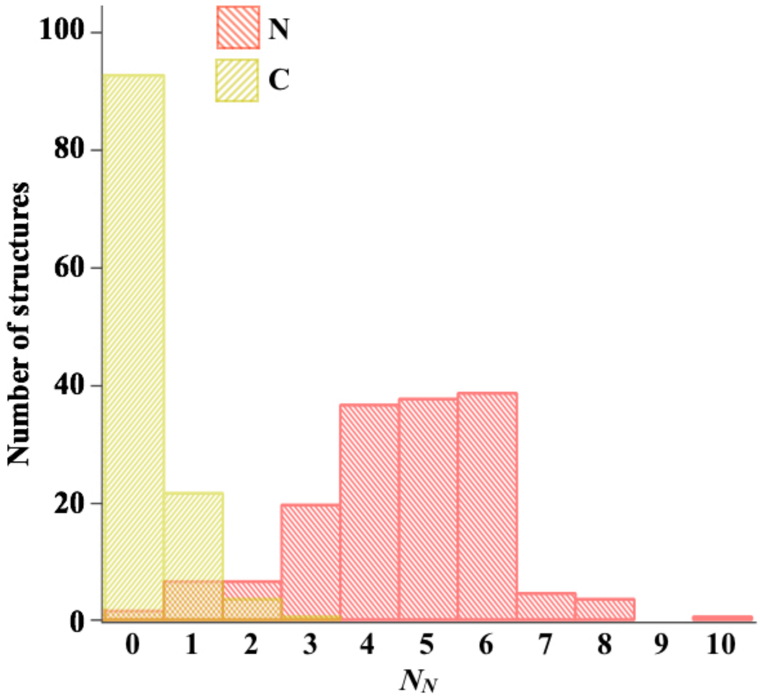

Fig. S3. Histogram summarizing the number of nerve-positive prediction points (N_N) on individual peripheral nerves (N: red) and connective tissues (C: yellow). The total number of peripheral nerves and connective tissues is 160 and 120, respectively.

Table S1. Results of nerve tissue detection using mean spectra at individual nerve and connective tissues. Results of two different procedures are shown.

Spectral treatment	Averaging, then preprocessing	Preprocessing, then averaging
True positive	148	145
False negative	10	13
True negative	119	120
False positive	1	0
Sensitivity (%)	93.7	91.8
Specificity (%)	99.2	100
Accuracy (%)	96.0	95.3
